# Competitions for Hospital Buildings

**Published:** 1912-03-30

**Authors:** A. Saxon Snell

**Affiliations:** 9 Bentinck Street, Manchester Square, W.


### THE EDITOR'S LETTER-BOX.

### COMPETITIONS FOR HOSPITAL BUILDINGS.

*To the Editor of* The Hospital.

Sir,—Your correspondent "Hospital Medical Officer" is indignant because he finds evidence in my letter to you of a "spirit of prejudice and exclusiveness." I am really sorry that anything I have written should convey that impression; but frankly I think a more careful reading of my letter will convince him that he does me an injustice. On closer acquaintance—which I hope for in the future—I am sure we shall find ourselves very much in agreement upon the relations which should obtain between medical officer and architect in the arrangement of a hospital, which the one has to administer and the other to design.

I have consistently maintained that the architect who attempts to build a hospital without reference to the views of physicians, surgeons, and matrons will achieve no progress in design, and courts failure in his building. On the other hand, the medical man who thinks that an architect is, or should be, a mere draughtsman, and that doctors alone know anything about the administration of a hospital, is guilty of that very "spirit of prejudice and exclusiveness" which I deprecate as strongly as "Hospital Medical Officer."

The method of conducting a competition, even in connection with a hospital, is, however, a matter upon which a medical man's opinion is of no greater value than that of any other intelligent man. It is purely—or perhaps I should say mainly—an architectural question.

Few, even of intelligent men, realise to what an extent the architectural profession is taxed under the competition system in time, energy, and money; and we who suffer must have the last word upon the terms under which we will compete.—Yours faithfully,

9 Bentinck Street, A. Saxon Snell.

Manchester Square, W., March 26.

